# An engineered autotransporter-based surface expression vector enables efficient display of Affibody molecules on OmpT-negative *E. coli* as well as protease-mediated secretion in OmpT-positive strains

**DOI:** 10.1186/s12934-014-0179-z

**Published:** 2014-12-30

**Authors:** Filippa Fleetwood, Ken G Andersson, Stefan Ståhl, John Löfblom

**Affiliations:** Division of Protein technology, School of Biotechnology, KTH Royal Institute of Technology, Stockholm, Sweden

**Keywords:** Affibody molecule, Bacterial display, Directed evolution, Combinatorial protein engineering, AIDA-I, Autotransporter, FACS, Secreted protein production, *E. coli*, Phage display

## Abstract

**Background:**

Cell display technologies (e.g. bacterial display) are attractive in directed evolution as they provide the option to use flow-cytometric cell sorting for selection from combinatorial libraries. The aim of this study was to engineer and investigate an expression vector system with dual functionalities: i) recombinant display of Affibody libraries on *Escherichia coli* for directed evolution and ii) small scale secreted production of candidate affinity proteins, allowing initial downstream characterizations prior to subcloning. Autotransporters form a class of surface proteins in Gram-negative bacteria that have potential for efficient translocation and tethering of recombinant passenger proteins to the outer membrane. We engineered a bacterial display vector based on the *E. coli* AIDA-I autotransporter for anchoring to the bacterial surface. Potential advantages of employing autotransporters combined with *E. coli* as host include: high surface expression level, high transformation frequency, alternative promoter systems available, efficient translocation to the outer membrane and tolerance for large multi-domain passenger proteins.

**Results:**

The new vector was designed to comprise an expression cassette encoding for an Affibody molecule, three albumin binding domains for monitoring of surface expression levels, an Outer membrane Protease T (OmpT) recognition site for potential protease-mediated secretion of displayed affinity proteins and a histidine-tag for purification. A panel of vectors with different promoters were generated and evaluated, and suitable cultivation conditions were investigated. The results demonstrated a high surface expression level of the different evaluated Affibody molecules, high correlation between target binding and surface expression level, high signal-to-background ratio, efficient secretion and purification of binders in OmpT-positive hosts as well as tight regulation of surface expression for the titratable promoters. Importantly, a mock selection using FACS from a 1:100,000 background yielded around 20,000-fold enrichment in a single round and high viability of the isolated bacteria after sorting.

**Conclusions:**

The new expression vectors are promising for combinatorial engineering of Affibody molecules and the strategy for small-scale production of soluble recombinant proteins has the potential to increase throughput of the entire discovery process.

**Electronic supplementary material:**

The online version of this article (doi:10.1186/s12934-014-0179-z) contains supplementary material, which is available to authorized users.

## Background

Protein engineering using combinatorial libraries is a well-established approach for selection of specific affinity proteins (e.g. recombinant antibody fragments). Selections from such libraries are commonly performed using various display technologies (e.g. phage display), creating a physical link between phenotype and genotype [1]. Cell surface display technologies (e.g. yeast and bacterial display) have several properties that are suitable for library applications, such as the convenience of using self-amplifying living cells [2]. The large particle size combined with a multivalent display format is particularly important since it allows for single cells to be sorted using fluorescence-activated cell sorting (FACS), enabling real-time visualization of the selection process, and quantitative isolation of high-affinity binders [3]. This is not possible using for example phage or ribosome display. We have previously developed a Gram-positive bacterial display technology based on expression of recombinant proteins on the surface of *Staphylococcus carnosus* [4,5]. The selection system has been successfully employed for generation of high-affinity single domain antibodies [6] and other alternative affinity proteins, including Affibody molecules [4,7-9]. Affibody molecules are based on a small three-helical domain (58 aa) with advantageous properties such as high thermal stability, rapid and reversible folding, high solubility, no cysteines, flexible pharmacokinetics and high tolerance for multiple mutations [10-12]. In a number of previous studies, a wide array of specific Affibody molecules with high affinity for various antigens has been selected using phage display, Gram-positive bacterial display and ribosome display [4,7,13]. A common challenge with all microbial-displayed combinatorial libraries is to reach a sufficiently high number of transformants during library construction. Gram-negative bacteria, such as *Escherichia coli,* are typically demonstrating higher transformation frequencies than for example yeast or Gram-positive bacteria and have the same potential as phage display for creation of really large libraries [14-16]. *E. coli* also has a rapid growth rate [17] and is the most well-characterized host for expression of recombinant proteins and is hence attractive for library applications. In this study, we therefore sought to design and evaluate an *E. coli* display method for Affibody libraries to complement the existing toolbox of technologies for engineering of new Affibody molecules.

Several formats for display of recombinant proteins and peptides on *E. coli* have been described previously [18-26]. However, although *E. coli* has several valuable properties, one of the major challenges is to enable the recombinant protein of interest to successfully cross two membranes and tether to the outer membrane [27]. In Gram-negative bacteria, a natural solution for secreting proteins to the cell surface is the autotransporter system [28]. The secreted protein consists of an N-terminal signal peptide followed by the passenger protein and a C-terminal β-domain. The signal peptide mediates transport of the unfolded protein sequence through the inner membrane to the periplasm using the *sec* apparatus [29]. Transit through the periplasm and secretion through the outer membrane is a complex process, involving several chaperones [30-32]. Briefly, the β-domain is inserted into the outer membrane where it forms a β-barrel pore structure, through which the passenger protein is transported to the cell surface [30-32]. In addition to the efficient transport mechanism of passenger proteins through both membranes, autotransporters are attractive alternatives for surface display of recombinant proteins because of their reported high surface expression levels [33]. A high surface expression has potential to yield a large signal-to-background ratio in the flow cytometer, and consequently a more efficient sorting of positive variants. Various autotransporters have been used for the display of different recombinant proteins, including disulphide-containing proteins [32,34].

In this study, we used the autotransporter *Adhesin Involved in Diffuse Adherence* (AIDA-I), which is a natural plasmid-encoded autotransporter of some enteropathogenic *E. coli* strains [35-37]. Importantly, AIDA-I has previously been successfully used for display of several different recombinant passenger proteins, which was encouraging for our efforts [33]. Earlier studies include for example work on toxins [22], enzymes, [38-41], and small libraries for engineering of enzyme inhibitors [33,42]. The Z domain from staphylococcal protein A has also been successfully used as a model passenger protein of AIDA-I in a study on optimization of bioreactor cultivation conditions [43], as well as for signal amplification in an immunoaffinity biosensor assay [44]. However, these methods were not intended and not evaluated for combinatorial protein engineering by FACS, and lacked for example strategies for normalization of the surface expression level. The first example of a modified autotransporter in combinatorial protein engineering of new affinity proteins was reported by Skerra and coworkers, describing the work on sortings from an anticalin library that was displayed using the autotransporter EspP [23]. More recently, another group displayed a nanobody library in fusion to the autotransporter EhaA and selected binders using magnetic beads [24]. The previous work demonstrates the potential for autotransporters in library applications. However, we are not aware of any reported use of AIDA-I for combinatorial protein engineering of affinity proteins.

In this study, an expression vector for display on *E. coli* was designed and engineered to comprise a subcloning site for Affibody molecules in fusion to the AIDA-I autotransporter. The expression cassette also included a surface expression reporter tag for normalization of target binding signal against expression levels during FACS. We investigated various promoters as well as different parameters for expression to find suitable culturing conditions for the constructs. The most promising vector was used for surface display of a panel of Affibody molecules with diverse specificities and FACS-mediated selection from a mock library efficiently enriched target-binding bacteria from a non-binding background. Furthermore, a straightforward strategy for small-scale protein production of soluble candidates using the same expression vector was explored. In summary, we have designed and evaluated new expression vector systems for Affibody molecules, which are intended for both display of combinatorial libraries as well as small-scale production of soluble proteins. We believe the expression systems have potential to further advance the discovery process of future Affibody reagents.

## Results and discussion

### Design and construction of dual-purpose expression vector

**Figure 1:**
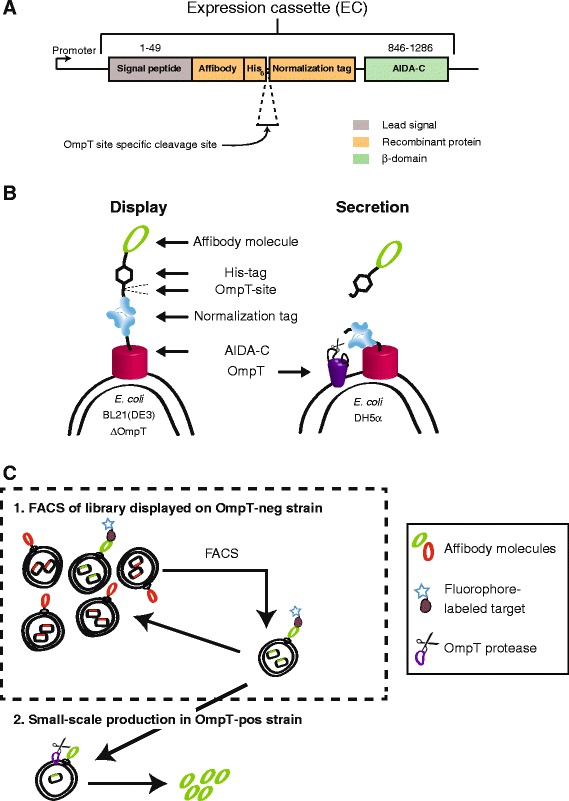
**Schematic illustration of the expression cassette, including the recombinant protein to be displayed on** ***E. coli*** **as well as the optional process for selection from displayed libraries or secreted production. A**. The expression cassette (EC) contains a promoter region, a signal peptide for translocation over the inner membrane, an Affibody molecule as binding protein, a His-tag for purification, an OmpT cleavage site for secretion, a surface expression reporter-tag for normalization and the AIDA-C for insertion into the outer membrane. AIDA-I numbering is according to the UniProt accession number Q03155 (http://www.uniprot.org). **B**. The recombinant fusion protein expressed in two different strains of *E. coli*. In an OmpT negative strain the recombinant protein remains tethered in the outer membrane and thus displayed on the bacterial surface, allowing for phenotype-genotype linkage and FACS. In an OmpT positive strain, OmpT will cleave the recombinant protein and release the Affibody molecule fused to a His-tag into the medium. **C**. Schematic drawing of a potential workflow using *E. coli* display for selecting Affibody molecules from libraries using FACS. After selection of new Affibody variants, the expression vector is transformed into an OmpT positive strain that results in protease-mediated secretion of candidates for downstream characterization.

**Figure 2:**
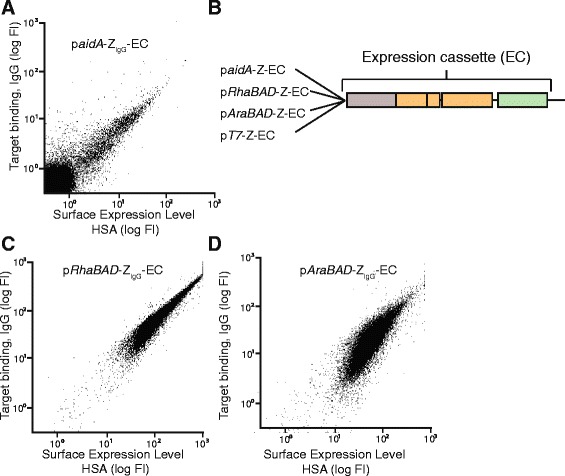
**Different inducible promoters compared to the constitutive** ***aidA*** **promoter for surface expression of recombinant protein. A**. Representative dot plot from flow-cytometric analysis of *E. coli* BL21 (p*aidA*-Z_IgG_-EC). Fluorescence intensity corresponding to IgG-binding on the y-axis and fluorescence intensity corresponding to surface expression level (HSA-binding) on the x-axis. **B**. The different inducible promoters *AraBAD*, *RhaBAD* and *T7* before the expression cassette (EC). **C**. Representative dot plot from flow-cytometric analysis of *E. coli* BL21 (p*RhaBAD*-Z_IgG_-EC). **D**. Representative dot plot from flow-cytometric analysis of *E. coli* BL21 (p*AraBAD*-Z_IgG_-EC).

In order to investigate the use of AIDA-I for display of Affibody molecules on the surface of *E. coli*, an expression vector was designed based on the pMK90 vector [45], encoding the aidA autotransporter gene (except the native passenger) under control of the constitutive *aidA* promoter [36] (Figure 1A). The expression vector encoded for a signal peptide from AIDA-I for translocation of the recombinant protein through the inner membrane, and the AIDA-I C-terminal β-domain for translocation of the recombinant protein through the outer membrane and display on the bacterial surface (Figure 1A). An IgG-specific Affibody molecule (Z_IgG_) was inserted between the signal peptide and the β-domain as model Affibody molecule for the study. An albumin binding protein (ABP) was fused on the C-terminal side of the Affibody and intended to function as a reporter tag for monitoring of the surface expression level of individual bacteria. It has previously been shown that normalization of target binding against surface expression level during FACS dramatically increases the ability to discriminate binders of different affinities [46]. ABP contains three albumin-binding domains derived from streptococcal protein G [47], and would hence serve as a long spacer that would minimize potential steric hindrance between the Affibody molecule and the target protein from the bulky cell surface (Figure 1A). As illustrated in Figure 1B-C, the expression vector was also engineered to potentially enable small-scale secretion-based production of selected affinity protein candidates after selection. An OmpT protease site was therefore introduced between the Affibody and the ABP, intended for protease-mediated release of the affinity protein into the medium during cultivation in an OmpT-positive *E. coli* strain (Figure 1A-C)*.* A His_6_-tag was also introduced to facilitate purification of the secreted product (Figure 1A-C). The dual-purpose expression vector was transformed to OmpT-negative *E. coli* BL21(DE3) and the functionality of the IgG-specific Affibody molecule as well as the ABP was investigated using flow-cytometric analysis. The functionality of the displayed Z_IgG_ was confirmed by the binding of biotinylated IgG (monitored by the binding of phycoerythrin-conjugated streptavidin to the biotinylated IgG, represented on the Y axis of the dot plot in Figure 2A). The IgG binding signal was normalized against the surface expression level by monitoring of the binding of Alexa Fluor 647-HSA conjugate to the ABP in the displayed construct (represented on the X axis in Figure 2A). The results demonstrated that the recombinant bacteria were indeed able to simultaneously bind fluorescently labeled IgG and HSA, indicating that both Z_IgG_ and ABP were functionally displayed on the *E. coli* outer membrane and the normalization strategy worked as intended (Figure 2A).

### Construction of expression vectors with inducible promoters

The natural AIDA-I promoter (*aidA*) is a constitutive promoter (Additional file 1: Table S1), which might result in problems with growth bias in future selections from bacterial-displayed libraries, as has been reported for other *E. coli* display technologies [48,49]. The expression cassette was therefore subcloned to three alternative vectors, containing inducible promoters (T7, *araBAD* and *rhaBAD*), and the new expression vectors were evaluated using flow cytometry (Figure 2B). The T7 promoter in combination with the BL21(DE3) strain is a well-established and strong promoter for overexpression of recombinant proteins, but is practically non-titratable (Additional file 1: Table S1) [50]. The *araBAD* and *rhaBAD* promoters are in contrast titratable promoters, enabling relatively precise control over the protein expression levels [51,52] (Additional file 1: Table S1). In general, high levels of displayed recombinant proteins are desirable for achieving a high fluorescent signal in the flow cytometer, but overexpression of recombinant membrane proteins might also cause issues with toxicity [48-50]. The surface expression level, monitored by the binding of HSA to ABP, and the binding of IgG to Z_IgG_, were analyzed by flow cytometry. Although bacteria with the T7 promoter were expressing recombinant protein on the surface, the vector was not further evaluated due to issues with low cell viability, substantially decreased growth rate and occasional loss of surface expression (data not shown). Similar problems have previously been reported with recombinant overexpression of membrane proteins and are likely due to saturation of the secretion pathway and connected toxicity [49,50]. The expression vectors containing the *araBAD* and the *rhaBAD* promoters demonstrated no toxicity-related issues and were therefore used in the further investigations (Figure 2C). Flow-cytometric analysis of non-induced controls yielded dramatically lower fluorescent signals, indicating that the observed fluorescence from the induced samples originated from specific interactions between recombinant Affibody molecules and fluorescently labeled target proteins on the bacterial surface (Additional file 2: Figure S1A).

### Evaluation of cultivation conditions

Next, we explored the range of suitable cultivation conditions using the two vectors that resulted in appropriate surface expression of the recombinant Affibody molecules. Induction time and temperature were evaluated by flow-cytometric analysis after induction in LB medium for 1 h, 3 h, 6 h and 16 h, at 25°C, 30°C and 37°C, respectively (Additional file 3: Figure S2A). The effect of inductor concentration was evaluated using different concentrations of L-arabinose and L-rhamnose (0.01%, 0.1%, 0.2%, 0.4%, 0.6%, 0.8% and 1.0%) (Additional file 3: Figure S2B). The screening revealed that both vectors should be induced for at least 3 hours for sufficient surface expression and no dramatic differences in expression were observed between 25°C, 30°C and 37°C. Still, induction at higher temperatures yielded in general a somewhat increased surface expression level (Additional file 3: Figure S2A). It should also be noted that the higher expression at 37°C for arabinose-induced cultures resulted in more non-expressing bacteria, which is an indication of overexpression-induced toxicity (Additional file 3: Figure S2A). The evaluation of various inductor concentrations demonstrated that 0.1% of either arabinose or rhamnose was sufficient for surface display of Affibody molecules (Additional file 3: Figure S2B). Under these conditions, the cell population was relatively uniform and well separated from the background, and the non-expressing population was in general small (Additional file 3: Figure S2). A clear correlation between IgG-binding fluorescence intensity and surface expression level was also observed, suggesting that the distribution in target-binding signal was mainly the result from variation in surface expression level among different cells (Additional file 3: Figure S2). It should be noted that the *rhaBAD* promoter requires addition of glucose to the medium for tight regulation, which might complicate extended assays that have a potential risk of glucose depletion [52]. Hence, to minimize such problems in future sortings of bacterial libraries, we used the *araBAD* promoter in the evaluations of the technology for isolation of bacterial clones with FACS.

### Estimation of display level

An estimation of the number of displayed recombinant proteins per cell was performed by a direct comparison to staphylococcal cells displaying Z_IgG_ on the surface [4]. The display level of recombinant staphylococci has previously been determined to around 10,000 copies per cell [53]. Comparison of the Z_IgG_-displaying *E. coli* to the staphylococci using flow cytometry revealed a higher mean fluorescence intensity for the recombinant *E. coli*, indicating that the display level was >10,000 copies per cell on average (Additional file 2: Figure S1B).

### Enzymatic detection of surface display

In order to confirm the display of the recombinant protein on the *E. coli* surface using a non-flow cytometric approach, an enzymatic assay was performed. Cells displaying Z_IgG_ were labeled with biotinylated IgG, followed by HRP-conjugated streptavidin. The IgG binding was detected by addition of substrate, and the absorbance was measured at 370 nM. Non-induced cells as well as cells labeled with only secondary reagents were included as negative controls. Almost a 4-fold higher binding signal was observed for the IgG-displaying *E. coli* cells compared to the negative controls, hence supporting the results from the flow-cytometric analysis (Additional file 4: Figure S3).

### Display of Affibody molecules with different target specificities

**Figure 3:**
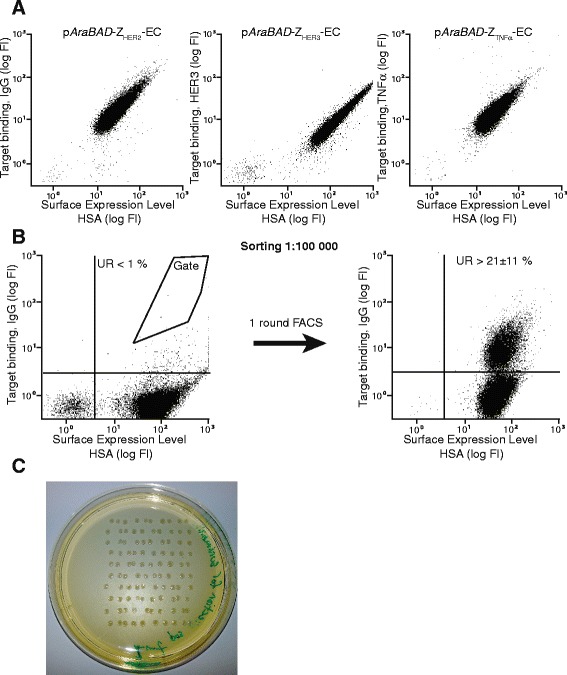
**Surface expression of different Affibody molecules on** ***E. coli*** **and fluorescence-activated cell sorting from spiked library. A**. Representative dot plots from flow-cytometric analysis of *E. coli* BL21 (p*AraBAD*-Z_HER2_-EC), (p*AraBAD*-Z_HER3_-EC) and (p*AraBAD*-Z_TNFα_-EC), respectively. Fluorescence intensity corresponding to target-binding on the y-axis and fluorescence intensity corresponding to surface expression level (HSA-binding) on the x-axis. **B**. Representative dot plots from flow-cytometric sorting of a 1:100,000 mix of *E. coli* BL21 (p*AraBAD*-Z_IgG_-EC) in a background of *E. coli* BL21 (p*AraBAD*-Z_HER2_-EC). Leftmost dot plot shows the mixed population before FACS with the sorting gate indicated in the dot plot. Rightmost dot plot shows the enriched bacteria after sorting and overnight growth. The percentage (mean ± standard deviation) of events in the upper right (UR) quadrant is indicated in the dot plots. The entire experiment was performed in duplicates on different days, using freshly prepared mixtures of bacteria expressing Z_HER2_ and Z_IgG_, and freshly prepared reagents. **C**. Viability test after FACS. Bacterial colonies after sorting 100 (10 x 10 pattern) single bacterial cells directly on a plate containing semi-solid medium with antibiotics and overnight incubation at 37°C. The viability assay was performed in triplicates on different days.

The flow-cytometric analysis demonstrated that the IgG-specific Affibody molecule was well expressed on the *E. coli* surface and retained its binding capacity to IgG. To verify that other Affibody molecules with diverse specificities and sequences also could be functionally displayed on the bacteria, the genes encoding three unrelated Affibody molecules (targeting TNF-α, HER3 and HER2, respectively) were subcloned into the expression vector and transformed to *E. coli* BL21 (DE3). Bacteria expressing the three different Affibody molecules were analyzed by flow cytometry and the assay demonstrated that all three were displayed at a high level on the surface and recognized their respective targets (Figure 3A). The results thus suggested that the display system was suitable for display of Affibody molecules with different target specificities, which was encouraging for future library applications.

### Investigation of enrichment factor by FACS

Flow-cytometric sorting was thereafter performed in order to investigate the ability to enrich target-binding bacteria from a large non-binding background population. Bacteria displaying Z_IgG_ on the surface were mixed at a ratio of 1:100,000 with cells displaying the HER2-specific Affibody molecule (Z_HER2_). For enrichment of Z_IgG_-displaying cells, one round of flow-cytometric cell sorting was performed using labeled IgG (Figure 3B). Sorted cells were collected and amplified by cell growth over night followed by determination of the enrichment using flow cytometry. The analysis demonstrated that the binding population in the upper right quadrant had increased to approximately 21 ± 11%, corresponding to an enrichment factor of around 20,000-fold in one cycle (Figure 3B).

### Investigation of cell viability after flow-cytometric cell sorting

Multiple sorting rounds are usually required for enrichment of high-affinity binders from large libraries and the possibility to amplify isolated clones by cell growth in between the cycles greatly facilitates the engineering process. However, high-speed cell sorting subjects the particles in the flow to relatively high shear stress, which might influence the viability of the bacteria. The degree of viability of the bacterial cells was hence investigated by high-speed sorting of recombinant *E. coli* directly onto plates with semi-solid medium, containing selective antibiotics. After overnight incubation, the number of formed colonies was counted and the results revealed that around 90% (87 ± 9%) of sorted bacteria were viable after FACS and able to grow into colonies (Figure 3C and Additional file 2: Figure S1C). Together with the high enrichment factor, the results from the viability assay suggested that the *E. coli* display technology has a high potential for future engineering of new Affibody molecules by FACS.

### Evaluation of OmpT-mediated release and small-scale purification of displayed recombinant proteins

**Figure 4:**
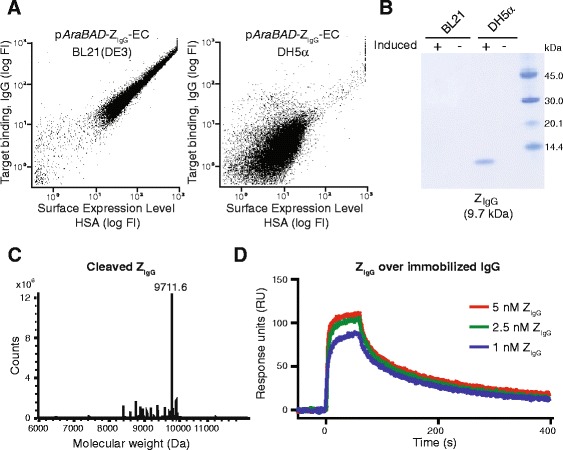
**Evaluation of OmpT-mediated release and small-scale purification of displayed Affibody molecule. A**. Flow-cytometric analysis for comparison of surface expression of recombinant proteins between p*AraBAD*-Z_IgG_-EC in OmpT-negative *E. coli* BL21 and p*AraBAD*-Z_IgG_-EC in OmpT-positive *E. coli* DH5α. **B**. SDS-PAGE showing IMAC-purified supernatants from p*AraBAD*-Z_IgG_-EC in OmpT-negative *E. coli* BL21 and p*AraBAD*-Z_IgG_-EC in OmpT-positive *E. coli* DH5α. Supernatants from non-induced samples, indicated with (−), were included as controls. **C**. Mass spectrum from ESI-MS analysis of IMAC-purified supernatants from p*AraBAD*-Z_IgG_-EC in OmpT-positive *E. coli* DH5α. Theoretical molecular weight of the OmpT-cleaved Z_IgG_-His_6_ is 9712 Da. **D**. SPR-based biosensor analysis on the IMAC-purified Z_IgG_-His_6_. Response units (RU) on the y-axis and time on the x-axis. Representative sensorgrams from injection of Z_IgG_-His_6_ at three different concentrations over human IgG immobilized on the chip surface. Injections were performed in duplicates.

Production and purification of soluble protein is necessary for downstream characterization of candidates selected from microbial-displayed libraries, and usually requires subcloning to a new expression vector. To potentially circumvent this step and improve the throughput of the entire protein engineering process, we included a His_6_-tag and an OmpT site for release of the affinity protein into the medium upon expression in OmpT positive strains (Figure 1)*.* The His_6_-tag was included to enable straightforward purification of the released affinity protein from the medium using IMAC. The efficiency of OmpT digestion for release of displayed protein into the medium was evaluated by transformation of the display vector into the OmpT positive strain *E. coli* DH5α (Invitrogen). Flow-cytometric analysis showed a dramatic reduction in target-binding signal in OmpT-positive bacteria, indicating that the Z_IgG_ had been proteolytically processed and released into the medium (Figure 4A). The surface expression level signal (monitored by the binding of fluorescently labeled HSA to ABP) was also somewhat reduced, potentially due to unspecific OmpT cleavage. The supernatant was purified using IMAC and SDS-PAGE revealed one band corresponding to the theoretical size and no detectable contaminants (Figure 4B). No bands were observed from samples with OmpT-negative bacteria or the non-induced controls, indicating that the purified protein corresponded to the recombinant Affibody molecule and that it was secreted by OmpT-mediated proteolysis (Figure 4B). The purified protein was analyzed by ESI-LC/MS, confirming that the product had the expected molecular weight, indicating a specific proteolytic release (Figure 4C). The concentration was estimated using UV spectrophotometry and the yield was calculated to around 3 ± 0.5 mg/l shake flask cell culture, which is usually sufficient for initial downstream characterization of selected Affibody candidates prior to subcloning for large scale production of the most promising variants. Moreover, the binding of Z_IgG_ to IgG was analyzed in a biosensor assay to verify that the function of the protein was not affected by the proteolytic digestion and purification strategy. IgG was immobilized on the chip surface and purified Z_IgG_ was injected at different concentrations followed by monitoring of the response. The results from the assay showed retained binding capacity of the proteolytically released Affibody molecule, indicating that the described secretion approach might be valuable in the future for rapid production of selected candidates after FACS without the need for subcloning (Figure 4D). The successful OmpT-mediated release of functional Z_IgG_ also confirmed the display of the recombinant proteins on the bacterial surface.

## Conclusions

We have investigated the AIDA-I autotransporter for display of Affibody molecules on *E. coli* and demonstrated efficient FACS-mediated enrichment of bacteria expressing target-binding Affibody molecules from a non-binding background population. Moreover, we showed that the same expression vector could be used for efficient small-scale production of soluble proteins, which were secreted into the medium. Secretion was achieved by insertion of an OmpT recognition site into the construct, resulting in proteolytic release of recombinant protein from the cell surface in OmpT-positive strains. The secretion was investigated using flow cytometry, IMAC, SDS-PAGE and mass spectrometry and the results demonstrated that the strategy yielded pure soluble proteins of correct size and retained binding functionality at a yield of around 3 mg purified protein per liter cell culture. Although secretion of recombinant proteins to the medium is typically more complicated in Gram-negative bacteria compared to other hosts [54], it has been demonstrated in previous studies [22,55]. For example, Sevastsyanovich and co-workers used a serine protease autotransporter from Enterobacteriaceae for successful secretion of various proteins [55]. The mechanism for secretion in their system was based on intramolecular cleavage of the translocated protein and therefore required no additional protease [55]. However, although it might be more efficient for soluble secretion, it requires mutation of the cleavage site for achieving surface display. In contrast, the approach used in our study allowed for either display on the outer membrane or secretion of soluble product into the supernatant by simply switching host strain. Maurer and co-workers used OmpT for release of recombinant passenger protein into the supernatant [22]. However, unlike the system presented in this study, their method was not intended for combinatorial protein engineering and small-scale protein production for downstream characterization of selected variants. In combinatorial protein engineering applications, it has potential to increase the throughput of future selections and downstream characterizations of Affibody molecules. A reduction in surface expression level of ABP observed in the flow-cytometric analysis indicated that unspecific cleavage of OmpT might occur. However, this did not affect the purity or size of the cleaved Z_IgG_ detected by SDS-PAGE and mass spectrometry. The site for unspecific cleavage could potentially be mapped and mutated in order to avoid any problems for future applications.

Four different promoters were evaluated in the study and the results showed that the titratable rhamnose and arabinose promoters were highly suitable for recombinant expression of Affibody molecules on *E. coli*. Flow-cytometric analysis showed a high surface expression level and high correlation between target binding and surface expression level, suggesting that ABP is appropriate as normalization tag in this system. In total, the recombinant fusion protein comprised four independently folded domains (i.e. one Affibody and three albumin-binding domains) as well as a His-tag and an OmpT-site, confirming that the AIDA-I autotransporter is efficient for translocation and tethering of relatively large and multidomain proteins. The analysis also revealed a high signal-to-background ratio, which is promising for future sorting of libraries. Indeed, we observed around 20,000-fold enrichment after one cycle when sorting target-binding bacteria from a 1:100,000 background. In combinatorial protein engineering, high cell viability after sorting is important in order to avoid time-consuming re-transformation steps or extended sortings to reach sufficient oversampling. We sorted the recombinant *E. coli* directly onto semi-solid medium and the viability assay revealed that around 90% of sorted cells could form colonies. The high viability indicated that the integrity of the outer cell membrane was not severely affected by the display of our recombinant protein construct. The viability was higher than reported in other similar studies [23] and might potentially be the result of the non-toxic surface expression levels that are achievable using the titratable promoters. Skerra and coworkers have previously evaluated AIDA-I along with a few other autotransporters for display of anticalines, demonstrating more promising results for the EspP [23]. In this study, we showed that AIDA-I can be used for efficient display of Affibody molecules under the control of a tightly regulated and titratable promoter. We found that the cultivation conditions and the choice of promoter had an impact on display level and cell viability. For example, the use of the *aidA* promoter resulted in a considerably higher degree of non-displaying cells compared to inducible promoters, and the T7 promoter had a severe negative impact on the cell viability. The results also indicate that many different types of autotransporters will probably be suitable for recombinant display, but each might require an independent optimization of cultivation conditions.

In summary, we have demonstrated that the engineered expression vectors are efficient for surface display of various Affibody molecules on *E. coli* as well as for secreted production of soluble recombinant proteins. In combination with the high transformation frequency of *E. coli*, the AIDA-I-based system has potential to be a powerful future complement to existing methods (e.g. phage and staphylococcal display) for generation of new Affibody molecules.

## Methods

### Bacterial strains

*Escherichia coli* strain RR1∆M15 [56] and *E. coli* strain DH5α (Invitrogen, Carlsbad, CA) were used for subcloning work. *E. coli* strain BL21 (DE3) (Merck, Darmstadt, Germany) was used for cell surface display. *E. coli* DH5α (Invitrogen) was used for OmpT-mediated release of recombinant protein into the medium.

### Construction of expression vectors

DNA fragments encoding Z_IgG_, His_6_, OmpT cleavage site and ABP were subcloned into the vector pMK90 [45], containing parts of the aidA gene under control of the *aidA* promoter [36]. The native passenger has been deleted from pMK90, and the AIDA-I sequence comprises: the natural AIDA-I signal peptide (49 aa), a linker region (78 aa), and the entire β-barrel domain (440 aa) [45]. The Z_IgG_-His_6_ DNA fragment was amplified by PCR using Phusion polymerase (Finnzymes, Espoo, Finland). The reverse primer was designed to contain an OmpT cleavage site on the C-terminal side of the Z_IgG_ sequence, and a mutation of a lysine in position 49 to glutamine in order to delete a potential OmpT cleavage site in Z_IgG_. The pMK90 plasmid and the PCR-amplified Z_IgG_-His_6_-OmpT fragment were digested with *Xma*I and *Xba*I (New England Biolabs, Beverly, MA, USA). The plasmid and PCR fragment were ligated using a T4 DNA ligase (New England Biolabs), and transformed to *E. coli* DH5α cells (Invitrogen) by heat shock. Plasmids were prepared using a QIAprep minispin kit (Qiagen, GmbH, Hilden, Germany). Sequences were confirmed by sequencing using BigDye thermo cycle sequencing reactions (Applied Biosystems, Foster City, CA) and a DNA sequencer ABI Prism® 3730 Analyzer (Applied Biosystems).

The gene encoding the albumin-binding protein (ABP; three albumin-binding domains from streptococcal protein G) was amplified by PCR from pSCZ1 [4] using specific primers and Phusion polymerase (Finnzymes). The PCR fragment and the pMK90Z_IgG_HisOmpT vector were digested with *Xba*I (New England Biolabs). The vector was first dephosphorylated using Antarctic Phosphatase (New England Biolabs) and purified by phenol chloroform extraction. The dephosphorylated and purified vector and the ABP fragment were ligated using T4 DNA ligase (New England Biolabs). The ligated vector was transformed into *E. coli* DH5α electrocompetent cells (Invitrogen), and plasmids were prepared using a QIAprep minispin plasmid preparation kit (Qiagen). Two potential OmpT protease cleavage sites in the ABP sequence were mutated (K51Q and K126Q) using QuikChange multi mutation kit (Stratagene, Heidelberg, Germany) according to the supplier’s recommendations.

The expression cassette, containing sequences encoding AIDA-I, Z_IgG_, His_6_, OmpT site and ABP, was amplified by PCR using the pMK90-Z_IgG_-His_6_-OmpT-ABP as template, and was subcloned into the plasmids pET-26b(+) (Merck, Darmstadt, Germany), pBAD33 [51] and pRHA67K (Xbrane Bioscience, Stockholm, Sweden). Correct clones were transformed to *E. coli* BL21 (DE3) (Merck), or DH5α (Invitrogen) using heat shock. The new expression vectors were denoted p*AraBAD-*Z-EC, p*RhaBAD-*Z-EC, p*aidA-*Z-EC and p*T7-*Z-EC, respectively.

### Cultivation of recombinant E. coli

Colonies of *E. coli* BL21 (DE3) (Merck) containing the display vectors were inoculated to Luria Bertani (LB) medium containing appropriate antibiotics and grown for 16 hours at 37°C and 150 rpm. An aliquot of the culture was diluted 1:100 and cultivated at 37°C and 150 rpm until absorbance at 600 nm (A_600_) reached 0.5. Recombinant protein expression was induced by addition of appropriate inductor (0.6% L-arabinose for all experiments carried out after the evaluation of cultivation conditions), or no inductor for non-induced negative controls, and grown at 25–37°C for 3 – 16 hours.

### Flow-cytometric analysis

An aliquot of approximately 10^8^ recombinant bacteria was added to 1×PBSP (phosphate-buffered saline (PBS) with 0.1% Pluronic F108 NF surfactant (BASF Corporation, Mount Olive, NJ)), pelleted by centrifugation (15000 g, 6 min, 4°C), re-suspended in 22 nM biotinylated human polyclonal IgG, and incubated at room temperature with gentle mixing for 45 min. Cells were then washed with ice-cold 1xPBSP, re-suspended in 10 μg/ml streptavidin conjugated with R-Phycoerythrin (Invitrogen) and 150 nM Alexa Fluor 647-Human Serum Albumin (HSA) conjugate, and incubated on ice for 30 min. The samples were then washed with ice-cold 1×PBSP, resuspended in 1×PBSP, and analyzed using a Gallios™ flow cytometer (Beckman Coulter, Inc., Indianapolis, IN, USA).

### Investigation of cultivation conditions

*E. coli* BL21 (DE3) (Merck) cells containing the p*AraBAD-*Z-EC and p*RhaBAD-*Z-EC vectors, respectively, were cultivated as described above. Induction temperature and induction time were investigated by induction for 1 h, 3 h, 6 h or 16 h at 37°C, 30°C or 25°C, respectively. For each sample, a non-induced control was included. Different concentrations of L-arabinose and L-rhamnose were investigated by induction using various concentrations (0.01%, 0.1%, 0.2%, 0.4%, 0.6%, 0.8% and 1.0%). Surface expression level and target binding was analyzed for all samples by flow cytometry as described above and the experiments were performed in duplicates on different days using freshly prepared samples and reagents.

### Estimation of display level

For estimation of the display level, recombinant *S. carnosus* cells displaying Z_IgG_ were cultivated as previously described [4], and labeled for flow cytometry in parallel to *E. coli* cells displaying Z_IgG_ under the control of the *AraBAD* promoter, induced with 0.6% L-arabinose overnight at 25°C.

### Enzymatic detection of surface display

*E. coli* BL21 (DE3) (Merck) cells containing the p*AraBAD-*Z-EC vector were cultivated as described above, and induced with 0.6% L-arabinose at 25°C overnight. A non-induced sample was included as a negative control. Cells were labeled with biotinylated IgG as described for the flow-cytometric analysis, followed by streptavidin-HRP (Dako, Glostrup, Denmark) as a secondary reagent. Detection of IgG binding was performed by resuspending the cells in 3,3′, 5,5′ Tetramethylbenzidine (TMB) substrate (Sigma), and the absorbance was measured at 370 nm using a Fluostar Omega plate reader (BMG Labtech, Germany). The experiment was performed in triplicates.

### Display of Affibody molecules with different target specificities

DNA fragments encoding the Affibody molecules: Z_HER3_ (Z_05416_, [7]), Z_HER2_ (Z_0342_, [57]) and Z_TNFα_ (Z_TNFα-1_ [4]) were amplified by PCR, and digested with *Nhe*I and *Xma*I restriction enzymes (New England Biolabs). The digested fragments were ligated into the p*AraBAD-*Z-EC, digested with the same enzymes using T4 DNA ligase (New England Biolabs), and transformed into *E. coli* RR1∆M15 [56]. Plasmids were prepared using a Jetstar Plasmid Maxiprep Kit (Genomed, Bad Oeynhausen, Germany) and transformed into *E. coli* BL21 (DE3) (Merck). Flow-cytometric analysis was performed as described above, but using biotinylated HER3 (R&D Systems), HER2 (R&D Systems), and TNFα (R&D Systems), respectively. The experiment was performed in duplicates on different days using freshly prepared samples and reagents.

### FACS

Recombinant *E. coli* BL21 (DE3) (p*AraBAD-*Z_IgG_-EC) displaying Z_IgG_ (cultured and induced as above) were mixed at a ratio of 1:100 000 with *E. coli* BL21 (DE3) (p*AraBAD-*Z_HER2_-EC) displaying the Affibody molecule Z_HER2_. Labeling of approximately 10^8^ cells with IgG for flow cytometry was performed as described above. One round of sorting was performed using a MoFlo Astrios flow cytometer (Beckman Coulter). Sorted cells were collected in an eppendorf tube containing LB medium, and incubated at 37°C for 1 h before inoculation to LB supplemented with 0.05 mg/ml chloramphenicol and cultivation overnight, followed by induction and labeling for flow-cytometric analysis. The experiment was performed in duplicates on different days using freshly prepared samples and reagents. In addition, 100 cells were sorted using a MoFlo Astrios flow cytometer (Beckman Coulter) using typical library sorting settings directly onto a TBAB plate containing 0.02 mg/ml chloramphenicol, and incubated at 37°C overnight.

### Evaluation of OmpT-mediated release of Z_IgG_ into the medium

The expression vector p*AraBAD-*Z_IgG_-EC was transformed into OmpT positive *E. coli* DH5α (Invitrogen) using electroporation. OmpT negative *E. coli* BL21(DE3) containing p*AraBAD-*Z_IgG_-EC as well as non-induced cultures of both strains were included as controls in the experiment. Bacteria were cultivated and induced as described above. Cells were pelleted by centrifugation at 16000 g, 4°C for 10 min. The supernatant was filtered (0.45 μm) followed by immobilized metal ion affinity chromatography (IMAC) using PD-10 columns (GE Healthcare, Uppsala, Sweden) containing 6 ml of HisPur™ Cobalt Resin (ThermoScientific, Rockford, USA). After loading the supernatant, the column was washed with 18 ml wash buffer (15 mM imidazole, 50 mM Na(P) 300 mM NaCl), followed by elution using PBS containing 0.5 M imidazole. The imidazole was removed using PD-10 desalting columns (GE Healthcare) Protein concentration was estimated using UV-spectrophotometry for calculation of yield. The experiment was performed in duplicates on different days using freshly prepared samples and reagents. The eluate was analyzed by SDS-PAGE (BioRad, Berkley, CA, USA) and ESI-LC/MS using an Agilent 6520 ESI-Q-TOF LC/MS instrument (Agilent Technologies, Santa Clara, CA, USA). The binding of purified Z_IgG_ to IgG was analyzed in an SPR-based biosensor assay using a Biacore 3000 instrument (GE Healthcare). Human IgG was immobilized by amine coupling on a CM5 chip (GE Healthcare, Uppsala, Sweden), and 1 nM, 2.5 nM and 5 nM purified Z_IgG_ was injected for 200 s, and allowed to dissociate for 1800 s.

## Additional files

Additional file 1: Table S1. Properties of the evaluated promoters and vectors. Table listing properties of the evaluated expression vectors, including: original vector backbone, promoter, inductor compound, size (bp), selection marker and copy number. Additional file 2: Figure S1. A. Comparison of induced and non-induced *E. coli* cells containing the display vectors p*AraBAD*-Z_IgG_-EC and p*RhaBAD*-Z_IgG_-EC. Histograms show the IgG-binding signal. C. Viability test after FACS. Bacterial colonies after sorting 100 (10 x 10 pattern) single bacterial cells directly on a plate containing semi-solid medium with antibiotics and overnight incubation at 37°C. B. Estimation of expression level. Histograms showing the IgG-binding signal obtained from flow-cytometric analysis of staphylococcal cells (containing the staphylococcal display vector pSCZ_IgG_) as well as *E. coli* cells containing the display vector p*AraBAD*-Z_IgG_-EC. Additional file 3: Figure S2. Evaluation of different induction conditions. A. Representative dot plots from flow-cytometric analysis of different induction conditions for *E. coli* BL21 (p*AraBAD*-Z_IgG_-EC) and (p*RhaBAD*-Z_IgG_-EC), respectively. Induction temperature and induction time were investigated by induction for 1 h, 3 h, 6 h or 16 h at 37°C, 30°C or 25°C, respectively (indicated above the dot plots). B. Representative dot plots from flow-cytometric analysis of different inductor concentrations for *E. coli* BL21 (p*AraBAD*-Z_IgG_-EC) and (p*RhaBAD*-Z_IgG_-EC), respectively. Different concentrations of L-arabinose and L-rhamnose were investigated by induction using various concentrations: 0.01%, 0.1%, 0.2%, 0.4%, 0.6%, 0.8% and 1.0% (indicated above the dot plots). Fluorescence intensity corresponding to target-binding on the y-axis and fluorescence intensity corresponding to surface expression level (HSA-binding) on the x-axis. Additional file 4: Figure S3. Enzymatic detection of surface display. Histograms showing the IgG-binding signal obtained from Z_IgG_-displaying bacteria labeled with biotinylated IgG and HRP-conjugated streptavidin. The binding signal for each sample is presented as the absorbance at 370 nm normalized against the absorbance of cells labeled with only secondary reagents. A sample with non-induced bacteria was included as negative control. The experiment was performed in triplicates.
