# Supplementary material for: An engineered autotransporter-based surface expression vector enables efficient display of Affibody molecules on OmpT-negative E. coli as well as protease-mediated secretion in OmpT-positive strains
Source: Microb Cell Fact. 2014 Dec 30;13:179. doi: 10.1186/s12934-014-0179-z (PMC4304625; doi:10.1186/s12934-014-0179-z)
Supplement: Additional file 1: Table S1. — Properties of the evaluated promoters and vectors. Table listing properties of the evaluated expression vectors, including: original vector backbone, promoter, inductor compound, size (bp), selection marker and copy number. [file 12934_2014_179_MOESM1_ESM.pdf]

Additional file 2: Table S1. Properties of the evaluated promoters and vectors

Promoter	Vector backbone	Inductor	Size (bp)	Selection marker	Copy #	Comment
aidA promoter	pMK90	NA	5521	Ampicillin	Low	Constitutively expressed<br>Native promoter for AIDA-I
T7 promoter	pET-26B	IPTG	7694	Kanamycin	High	High expression level<br>Conventional promoter
RhaBAD promoter	pRha811	L-rhamnose	7230	Kanamycin	High	Tightly regulated<br>Fine-tuned expression using both repressive D-glucose and inducing L-rhamnose
AraBAD promoter	pBAD33	L-arabinose	7890	Chloramphenicol	Low	Tightly regulated<br>Low basal expression
