# Supplementary figures and images for: An engineered autotransporter-based surface expression vector enables efficient display of Affibody molecules on OmpT-negative E. coli as well as protease-mediated secretion in OmpT-positive strains
Source: Microb Cell Fact. 2014 Dec 30;13:179. doi: 10.1186/s12934-014-0179-z (PMC4304625; doi:10.1186/s12934-014-0179-z)

**A**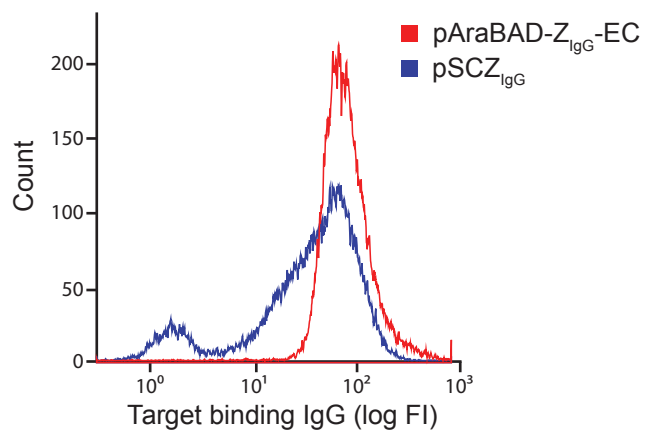**B**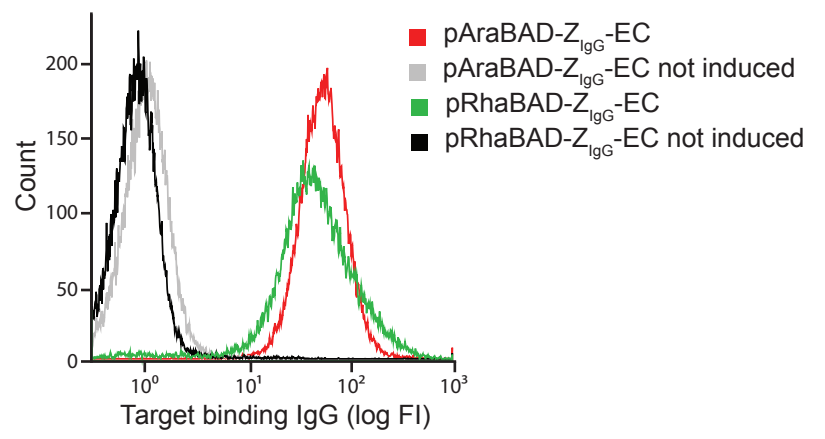**C**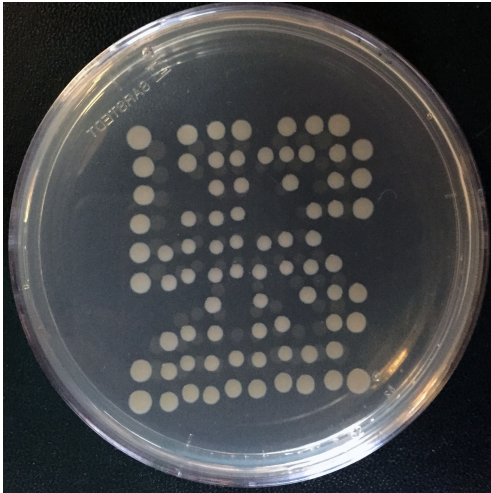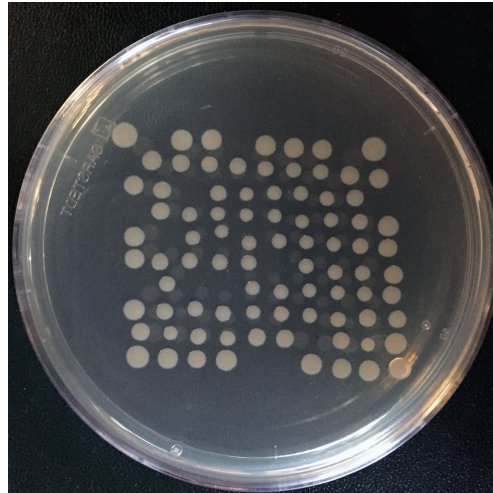

Supplement: Additional file 2: Figure S1. — A. Comparison of induced and non-induced E. coli cells containing the display vectors pAraBAD-ZIgG-EC and pRhaBAD-ZIgG-EC. Histograms show the IgG-binding signal. C. Viability test after FACS. Bacterial colonies after sorting 100 (10 x 10 pattern) single bacterial cells directly on a plate containing semi-solid medium with antibiotics and overnight incubation at 37°C. B. Estimation of expression level. Histograms showing the IgG-binding signal obtained from flow-cytometric analysis of staphylococcal cells (containing the staphylococcal display vector pSCZIgG) as well as E. coli cells containing the display vector pAraBAD-ZIgG-EC. [file 12934_2014_179_MOESM2_ESM.pdf]

**A**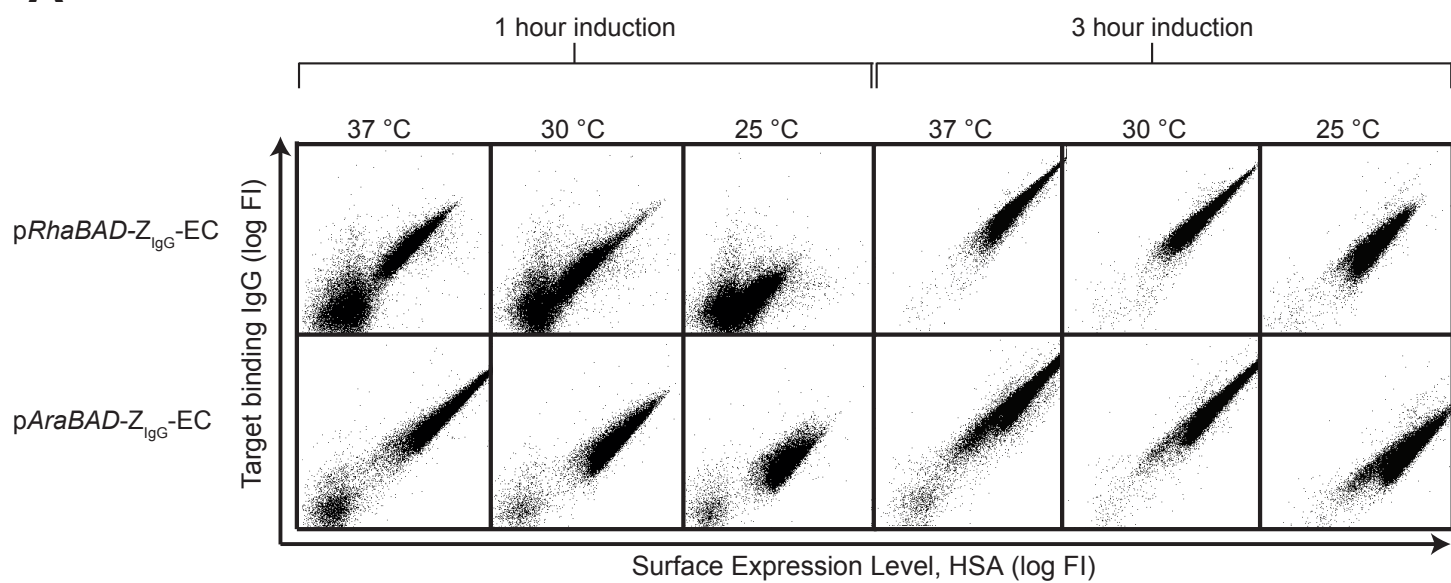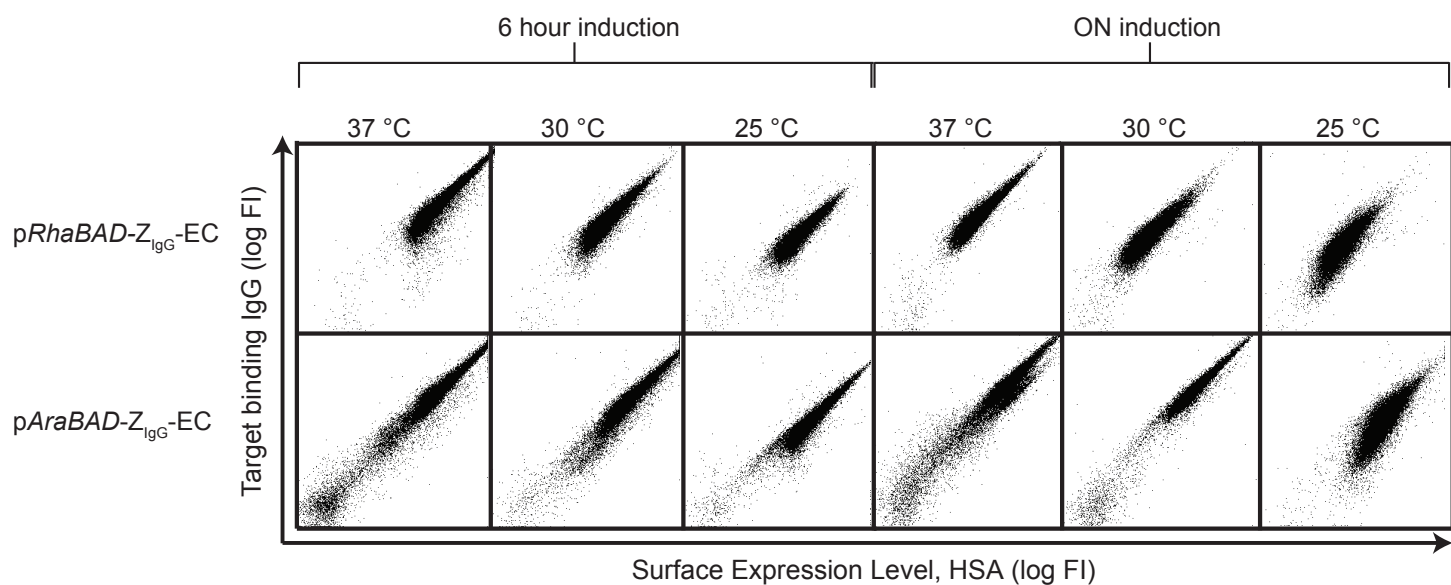**B**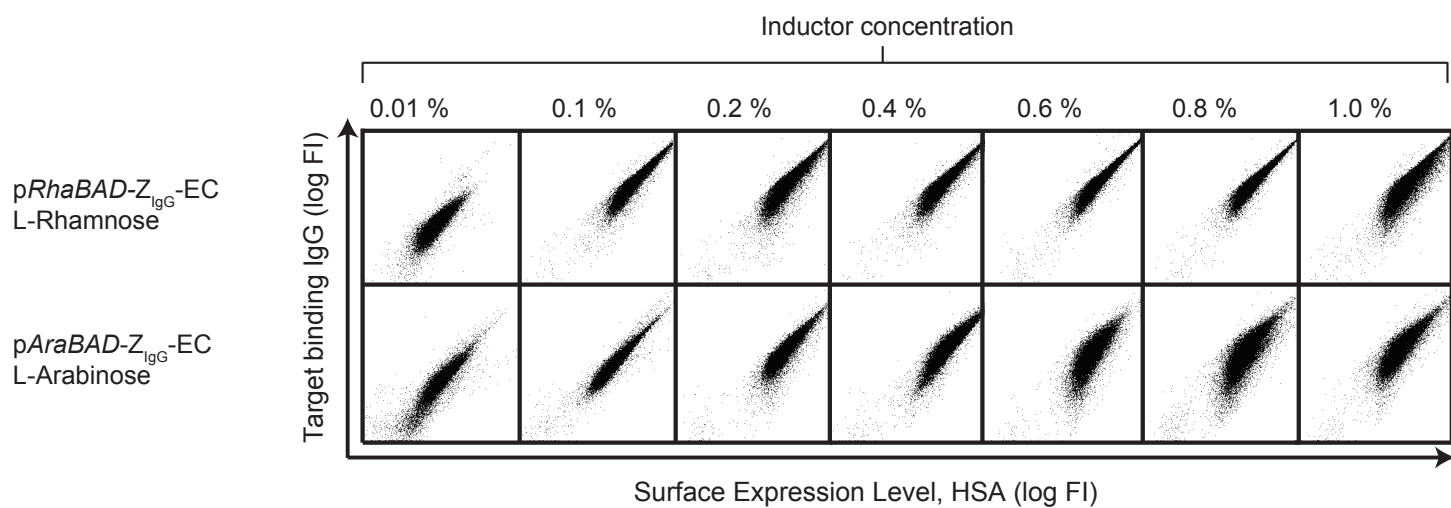

Supplement: Additional file 3: Figure S2. — Evaluation of different induction conditions. A. Representative dot plots from flow-cytometric analysis of different induction conditions for E. coli BL21 (pAraBAD-ZIgG-EC) and (pRhaBAD-ZIgG-EC), respectively. Induction temperature and induction time were investigated by induction for 1 h, 3 h, 6 h or 16 h at 37°C, 30°C or 25°C, respectively (indicated above the dot plots). B. Representative dot plots from flow-cytometric analysis of different inductor concentrations for E. coli BL21 (pAraBAD-ZIgG-EC) and (pRhaBAD-ZIgG-EC), respectively. Different concentrations of L-arabinose and L-rhamnose were investigated by induction using various concentrations: 0.01%, 0.1%, 0.2%, 0.4%, 0.6%, 0.8% and 1.0% (indicated above the dot plots). Fluorescence intensity corresponding to target-binding on the y-axis and fluorescence intensity corresponding to surface expression level (HSA-binding) on the x-axis. [file 12934_2014_179_MOESM3_ESM.pdf]

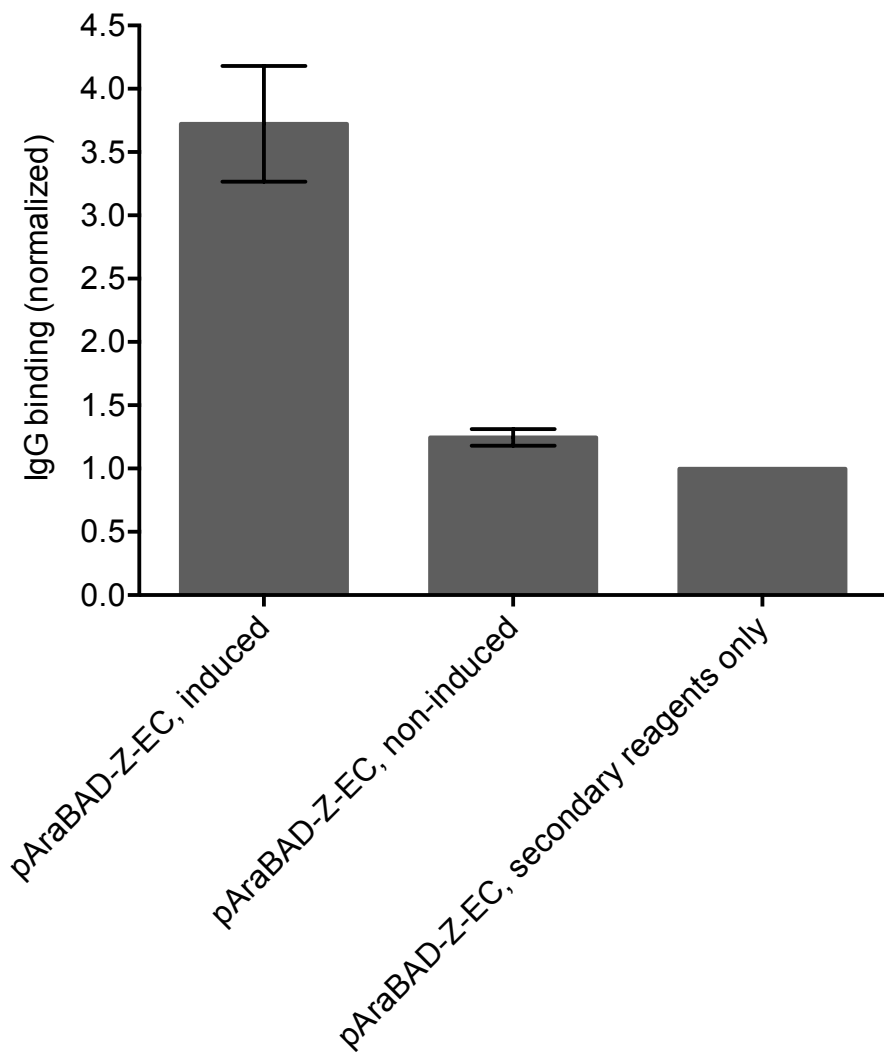

Supplement: Additional file 4: Figure S3. — Enzymatic detection of surface display. Histograms showing the IgG-binding signal obtained from ZIgG-displaying bacteria labeled with biotinylated IgG and HRP-conjugated streptavidin. The binding signal for each sample is presented as the absorbance at 370 nm normalized against the absorbance of cells labeled with only secondary reagents. A sample with non-induced bacteria was included as negative control. The experiment was performed in triplicates. [file 12934_2014_179_MOESM4_ESM.pdf]
